# Tumour cell-activated platelets modulate the immunological activity of CD4^+^, CD8^+^, and NK cells, which is efficiently antagonized by heparin

**DOI:** 10.1007/s00262-022-03186-5

**Published:** 2022-03-14

**Authors:** Lukas M. Gockel, Katrin Nekipelov, Vito Ferro, Gerd Bendas, Martin Schlesinger

**Affiliations:** 1 Pharmaceutical Institute, Pharmaceutical and Cell Biological Chemistry, University of Bonn, 53121 Bonn, Germany; 2 School of Chemistry and Molecular Biosciences, The University of Queensland, Brisbane, QLD 4072 Australia

**Keywords:** Cancer, Heparin mimetics, Immunomodulation, LMWH, Platelets, T cells

## Abstract

Platelets, key players in haemostasis, are progressively investigated with respect to their role in immunity and inflammation. Although the platelet support to haematogenous cancer cell metastasis has been the subject of multiple studies, their impact on anti-cancer immunity remains unaddressed. Here, we investigated the immunomodulatory potential of platelets upon their activation by MDA-MB-231 breast cancer cells in various in vitro approaches. We provide evidence that platelets as well as their tumour cell-induced releasates increased the ratio of regulatory T cells, shaping an immunosuppressive phenotype in isolated CD4^+^ cultures. The influence on CD8^+^ T cells was assessed by detecting the expression of activation markers CD25/CD69 and release of cytolytic and pro-inflammatory proteins. Notably, the platelet preparations differentially influenced CD8^+^ T cell activation, while platelets were found to inhibit the activation of CD8^+^ T cells, platelet releasates, in contrast, supported their activation. Furthermore, the NK cell cytolytic activity was attenuated by platelet releasates. Low molecular weight heparin (LMWH), the guideline-based anticoagulant for cancer-associated thrombotic events, is known to interfere with tumour cell-induced platelet activation. Thus, we aimed to investigate whether, unfractionated heparin, LMWH or novel synthetic heparin mimetics can also reverse the immunosuppressive platelet effects. The releasate-mediated alteration in immune cell activity was efficiently abrogated by heparin, while the synthetic heparin mimetics partly outperformed the commercial heparin derivatives. This is the first report on the effects of heparin on rebalancing immunosuppression in an oncological context emerging as a novel aspect in heparin anti-tumour activities.

**Supplementary Information:**

The online version contains supplementary material available at 10.1007/s00262-022-03186-5.

## Introduction

With an estimated 10 million lethal outcomes in 2020, cancer is considered worldwide as a major cause of death, especially in Northern America and Europe, where it surpassed the cardiovascular diseases and ranks in the first place [1, 2]. In fact, about 60% of cancer deaths do not arise from primary tumours, but are associated with the development of metastases [3]. To form metastases, tumour cells invade the blood circulation and are disseminated to distant organs. On their way, circulating tumour cells are exposed to a hostile environment and, e.g., have to cope with the attack of immune cells. Thus, not only during haematogenous passage, evasion of host immunosurveillance is crucial for tumour survival and progression [4]. As the first cells encountered by cancer cells after entering the blood system, platelets are considered to support tumour cell survival during haematogenous metastasis. Upon activation by cancer cells, they form a protective cloak around the cancer cell, shielding them from shear forces or immunosurveillance by NK cells and supporting adhesion and subsequent extravasation of disseminating cancer cells [5–8].

Besides their crucial role in haemostasis, platelets are progressively considered as immune-competent cells participating in inflammation and immunity. Platelets were shown to directly contribute to anti-viral and anti-bacterial immunity by release of microbicidal factors, pathogen engulfment or aggregate formation [9–11]. In particular, by interaction with lymphocytes platelets are capable of affecting their immunological functions. For instance, platelets stimulated immunoglobulin production of B cells in co-culture experiments and support T cell activation and recruitment to the site of inflammation [12–15]. Recently, Rossiant and colleagues showed the adaptable role of platelets in onset and resolution of pulmonary inflammation by modulation of neutrophils, regulatory T cells (Tregs), and macrophages [16].

However, the multifaceted role of platelets in cancer immunity is not yet fully elucidated. Regarding the cellular level, natural killer cells (NK cells) and cytotoxic T lymphocytes identify and neutralize cancer cells efficiently and are at the frontline in the immune response against cancer cells. Together with CD4^+^ T cells, they can be functionally impaired by cancer cells themselves or the composition of the tumour microenvironment with strong contribution of adjacent platelets [17, 18].

Reasoned by the elevated incidence, low molecular weight heparins (LMWH) are the guideline based drug for prevention and therapy of venous thromboembolism in cancer patients [19]. Since some clinical trials observed additional survival benefits for LMWH treated patient subgroups, while others did not, there is an ongoing debate whether LMWH exerts anti-metastatic effects beside its anticoagulant properties [20–22]. Thus, several metastasis-related targets of heparin have been postulated and are currently in debate [23]. Although the interference of heparins in platelet activation as crucial steps of the metastatic cascade appears likely [24, 25], its impact on immunomodulating platelet activities remains elusive.

Recently, we showed that synthetic heparin mimicking polymers consisting of sodium 4-styrenesulfonate (SSS), potassium-3-sulfopropyl acrylate (SPA) and acrylic acid (AA) monomers kept up and even outmatched commercial heparins in interfering in the metastasis-associated platelet–tumour cell interaction [25]. Here, we focus on the immunomodulatory function of platelets with regard to Treg cell differentiation, CD8^+^ T cell activity as well as NK cell cytolytic activity in a tumour cell context. We show that heparins and heparin mimetics are able to block the platelet supernatant mediated Treg differentiation. Additionally, heparin mimetics reduced platelet-induced CD8^+^ T cell activation. Thus, our data reveal a novel immunological function of heparin in the platelet tumour cell communication, which warrants further investigations.

## Materials and methods

### Cell isolation

Buffy coats from healthy donors were obtained from the Institute for Experimental Hematology and Transfusion Medicine, University of Bonn, Medical Centre. PBMCs were isolated from buffy coats by density gradient centrifugation using Histopaque^®^-1077 (Sigma-Aldrich, Steinheim, Germany). Human CD4^+^ T cells and CD8^+^ T cells were extracted from buffy coats by positive selection using the StraightFrom Buffy Coat CD4 MicroBead Kit and the StraightFrom Buffy Coat CD8 MicroBead Kit (Miltenyi Biotec, Bergisch Gladbach, Germany), respectively.

### Platelet preparations

Platelet-rich plasma (PRP) from healthy donors was obtained from the Institute for Experimental Hematology and Transfusion Medicine, University of Bonn, Medical Centre. Platelets were centrifuged at 670 × g and RT for 10 min. The platelet pellet was resuspended in platelet buffer (10 mM HEPES, 140 mM NaCl, 3 mM KCl, 0.5 mM MgCl_2_, 5 mM NaHCO_3_, and 10 mM glucose) at a final concentration of 1.25 × 10^9^ plts/mL. The platelet suspension was supplemented with a final concentration of 1 mM Ca^2+^ and 0.5% plasma. This suspension was used in experiments or was further processed to platelet releasates. Platelet releasates were obtained by co-incubation of 1.25 × 10^9^ plts/mL with 3.125 × 10^4^ MDA-MB-231 breast cancer cells (ratio 40.000:1). After an activation time of 20 min, the platelet/cancer cell aggregates were removed by centrifugation at 10,000 × g, 4 °C for 10 min. Supernatants were collected and used as platelet releasates in the performed assays. In some cases, platelets were incubated with the indicated concentrations of the tested compounds for 30 min prior to activation. For this, platelets were treated with commercially available anticoagulants, unfractionated heparin (UFH) (Ratiopharm GmbH, Ulm, Germany), enoxaparin (Sanofi-Aventis Deutschland GmbH, Frankfurt, Germany), and fondaparinux (Aspen Pharma Trading Ltd, Dublin, Ireland) at adopted therapeutic concentrations of 3.125 U/mL, 3.125 U/mL, and 2.422 µg/mL, respectively. The heparin mimetics were investigated at final concentrations of 156.25 µg/mL.

### Cell culture

Human breast cancer cell line MDA-MB-231 (RRID:CVCL_0062, ATCC) was maintained in high-glucose Dulbecco’s Modified Eagle’s Medium (DMEM) supplemented with 10% FCS, 2 mM l-glutamine, 1 mM sodium pyruvate, 100 U/mL penicillin, and 100 µg/mL streptomycin (all from PAN Biotech, Aidenbach, Germany). Human PBMC, CD4^+^ T cells, CD8^+^ T cells, and K562 (RRID:CVCL_0004; ATCC) were cultured in RPMI-1640 medium supplemented with 10% FCS, 100 U/mL penicillin, and 100 µg/mL streptomycin (all from PAN Biotech).

### IL-10 ELISA

PBMCs were activated by stimulating antibody cocktail ImmunoCult™ Human CD3/CD28/CD2 T Cell Activator (Stemcell Technologies Inc., Vancouver, Canada) and 100 U/mL IL-2. 1.25 × 10^6^ PBMC/mL were spiked with 3.125 × 10^8^ plts/mL or the corresponding releasate on days 1 and 3, respectively. Cell culture supernatants were collected on day 5 and centrifuged at 6000 × g at 4 °C for 10 min to remove residual cells. Samples were investigated for their IL-10 levels applying the Human IL-10 Mini ABTS ELISA Development Kit (PeproTech Inc, Rocky Hill, NJ, the USA).

### Treg differentiation

CD4^+^ T cells were stimulated by ImmunoCult™ Human CD3/CD28/CD2 T Cell Activator at day 1. At days 1 and 3, platelets were added to CD4^+^ cells (6.25 × 10^7^ plts/mL and 2.5 × 10^5^ CD4^+^/mL), or the corresponding platelet releasate was used. Cells were collected at day 5 and investigated for CD25 and FoxP3 expression. Cells were washed once with staining buffer (1% BSA, 0.01% NaN_3_ in DPBS). To avoid unspecific binding, cells were blocked for 15 min with 3% BSA in DPBS. Anti-CD25 antibody (PerCP/Cyanine5.5 anti-human CD25 antibody, RRID:AB_2125478, BioLegend Inc, San Diego, CA, USA) was added and incubated for 30 min in the dark. Subsequently, cells were washed once with staining buffer and fixed in 150 µL fixation buffer (3% PFA, 0.1% saponin, and 0.5% Tween-20 in DPBS) for 30 min at 4 °C. Samples were washed once with permeabilization buffer (0.5% saponin, 0.5% Tween-20 in staining buffer). Next, cells were permeabilized in 75 µL permeabilization buffer for 30 min at 4 °C. Samples were spiked with anti-FoxP3 antibody (FITC anti-human FoxP3 antibody, RRID:AB_439752, BioLegend Inc.) diluted in 25 µL permeabilization buffer. After an incubation of 30 min at 4 °C, cells were washed twice with permeabilization buffer and finally resuspended in staining buffer. Samples were acquired using the Guava^®^ easyCyte HT 11 Flow Cytometer (Luminex Corporation, Austin, TX, the USA). Fluorescence Minus One (FMO) controls were conducted to set up the applied gates. Anti-mouse Igκ MACS^®^ Comp beads (Miltenyi Biotec) were used for compensation of fluorescence labelled antibodies. Data were evaluated using FlowJo™ v10.5.3 Software (RRID:SCR_008520, BD Life Sciences, Franklin Lakes, NJ, the USA). Applied gating is depicted in supplementary Fig. 1.

**Fig. 1:**
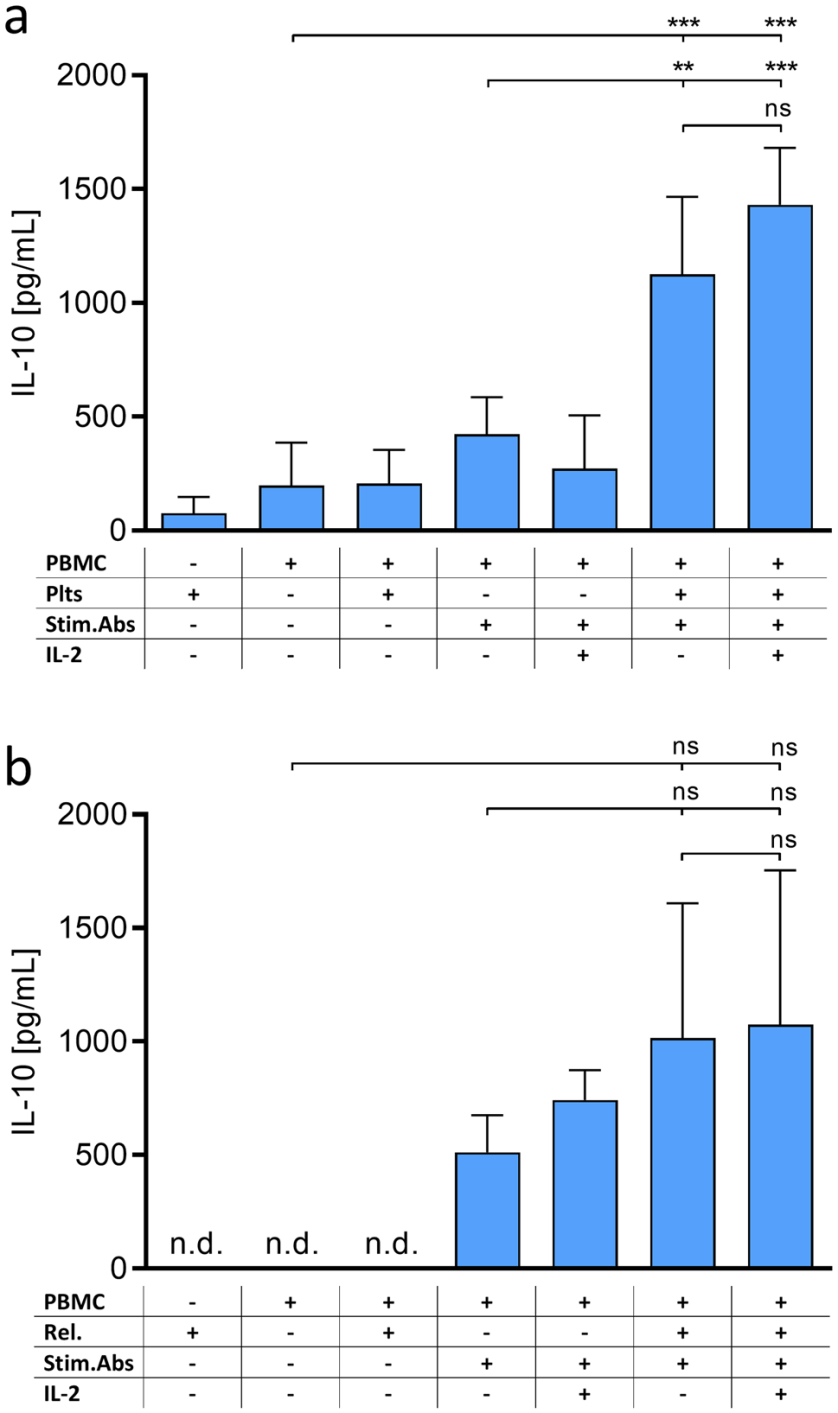
IL-10 release from human PBMCs upon activation (Stim. Abs.) and co-culture with **a** platelets (plts), or **b** platelet releasates (rel.). Data represent mean ± SD (*n* = 3) with n.d.: not detectable, ns: not significant, **: *p* < 0.01 and ***: *p* < 0.001, respectively

### CD8^+^ T cell activity

Human CD8^+^ T cells (2.5 × 10^5^ /mL) were stimulated at day 1 by ImmunoCult™ Human CD3/CD28/CD2 T Cell Activator and were spiked with 6.25 × 10^7^ platelets per mL or the corresponding platelet releasate at days 1 and 3. Cells were collected at day 5 and washed once with DPBS. Following, CD8^+^ T cells were stained for viability using Zombie Aqua™ (BioLegend Inc.). After 20 min incubation at RT, the staining was quenched with staining buffer. Subsequently, cells were centrifuged, resuspended and incubated for 15 min in a 3% BSA solution to block unspecific binding. After blocking, cells were stained with Pacific Blue anti-human CD8a antibody (RRID:AB_159443, BioLegend Inc.), PerCP/Cyanine5.5 anti-human CD25 antibody (RRID:AB_2125478, BioLegend Inc.), and FITC anti-human CD69 antibody (RRID:AB_314839, BioLegend Inc.), respectively. Cells were washed twice with staining buffer. Samples were measured in staining buffer using a Guava^®^ easyCyte HT 11 Flow Cytometer (Luminex). FMO controls were performed to set up the applied gates. For compensation of antibody-coupled dyes, anti-mouse Igκ MACS^®^ Comp beads (Miltenyi Biotec) were used. For compensation of Zombie Aqua™ dead cells were killed by an incubation at 70 °C for 12 min followed by 5 min on ice. Data were analysed using FlowJo™ v10.5.3 Software (BD Life Sciences). Gating strategy is shown in supplementary Fig. 2.

**Fig. 2:**
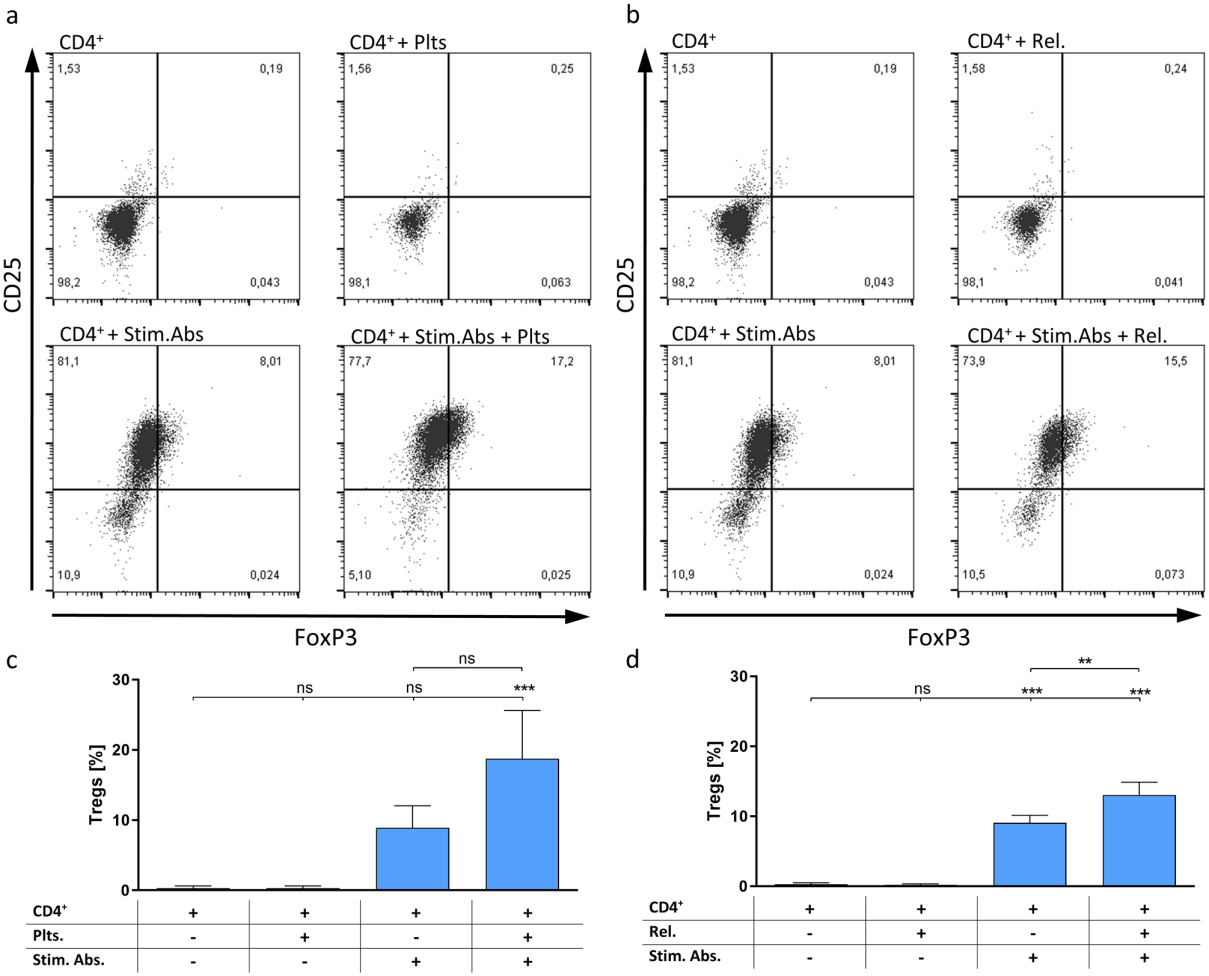
Determination of Treg induction in activated CD4^+^ cell populations (Stim. Abs.). Regulatory T cells were determined as CD25/FoxP3 double-positive population by flow cytometry. Representative dot plots are depicted for **a** platelets (plts) and **b** releasates (rel.). Summarized data for **c** platelets and **d** releasates are shown as mean ± SD (*n* = 3) with ns: not significant, **: *p* < 0.01 and ***: *p* < 0.001, respectively

### Multiplex analysis

Human CD8^+^ T cells were stimulated as described above. Cell culture supernatants were collected at day 5 and centrifuged at 10,000 × g for 5 min to remove residual cells. Supernatants were investigated for cytokine release from CD8^+^ T cells using the LEGENDplex™ Human CD8/NK Panel (BioLegend Inc.) according to the manufacturer’s instructions. Samples were diluted 1:100 prior to the analysis. Samples were acquired using a CytoFlex flow cytometer (Beckmann Coulter GmbH, Krefeld, Germany). Data evaluation was performed using the LEGENDplex™ Data Analysis Software (BioLegend Inc.).

### NK cell cytotoxicity

Human PBMCs were cultured for 24 h in the presence of platelet releasates in a 1:250 ratio. K562 cells were labelled using CFSE (BioLegend Inc.) and were co-cultured with PBMCs in a 50:1 Effector/Target (ET) ratio following the protocol published by Kandarian et al. [26]. Differing, 7-AAD (BioLegend Inc.) was used as live/dead staining. Samples were acquired by a Guava^®^ easyCyte HT 11 Flow Cytometer (Luminex) and evaluated using FlowJo™ v10.5.3 Software (BD Life Sciences). Applied gates and corresponding controls are depicted in supplementary Fig. 3a.

**Fig. 3:**
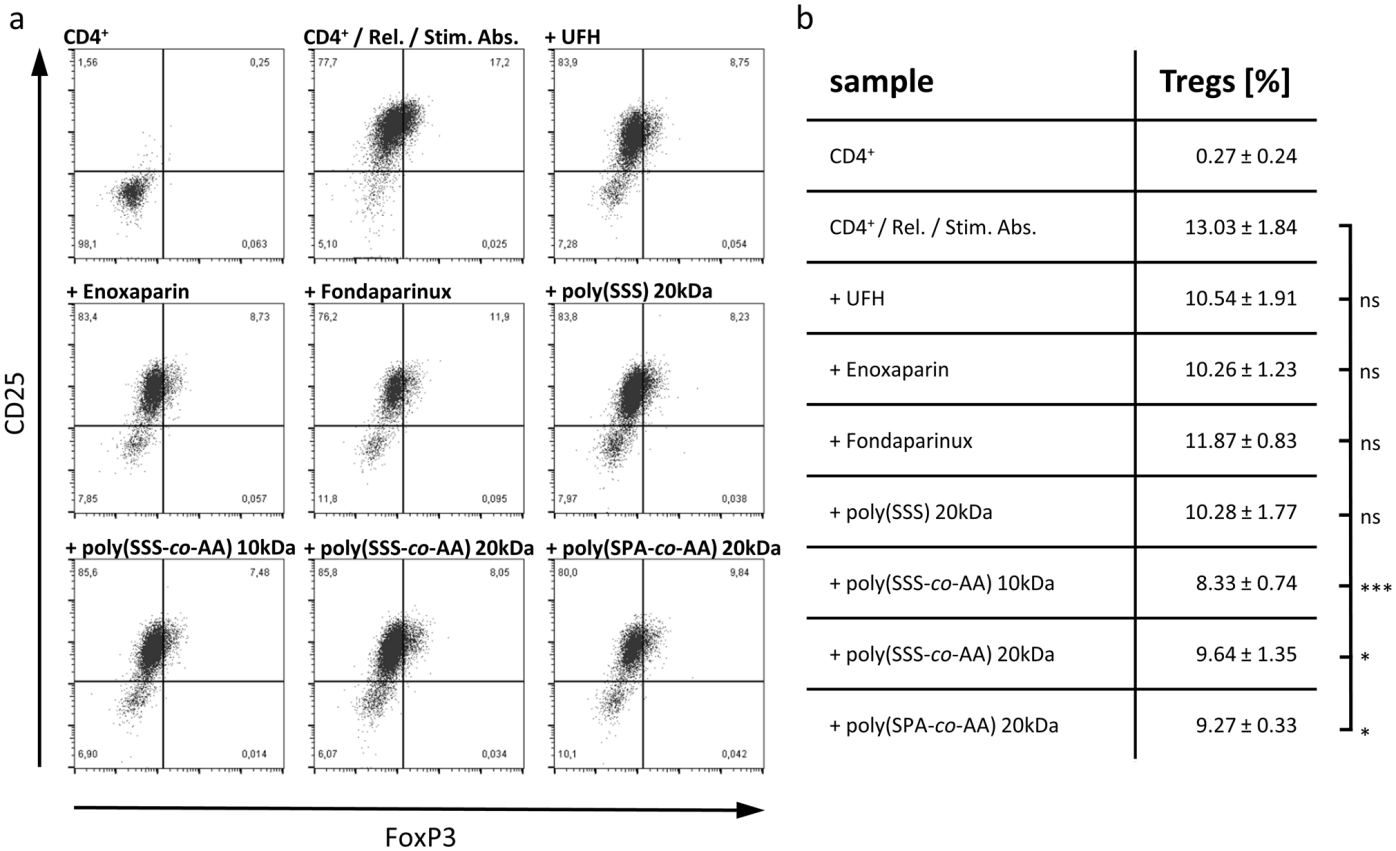
Influence of heparins and heparin mimetics on the capacity of platelet releasates (rel.) to induce regulatory T Cell (Treg) differentiation in activated CD4^+^ cell populations (Stim. Abs.). **a** CD25/FoxP3 dot plots are representatively shown for each tested compound. **b** summarized percentages for each compound are reported as mean ± SD (*n* = 3) with ns: not significant, *: *p* < 0.05, and ***: *p* < 0.001

### Statistical analysis

**Fig. 4:**
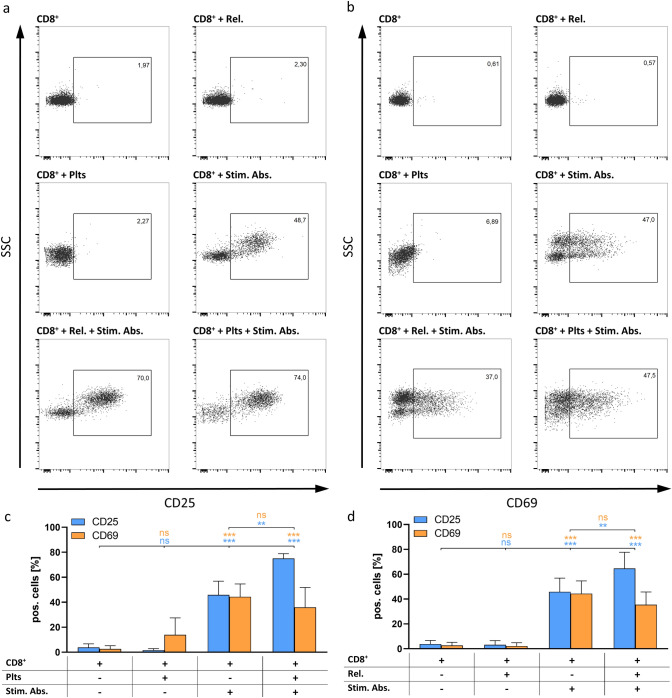
Flow cytometric analysis of CD25 and CD69 expression on activated CD8^+^ T cells. Representative dot plots are shown for **a** CD25 and **b** CD69. Summarized data for **c** platelets co-culture (plts) and **d** releasates co-culture (rel.) are shown as mean ± SD (*n* = 3) with ns: not significant, **: *p* < 0.01 and ***: *p* < 0.001, respectively

**Fig. 5:**
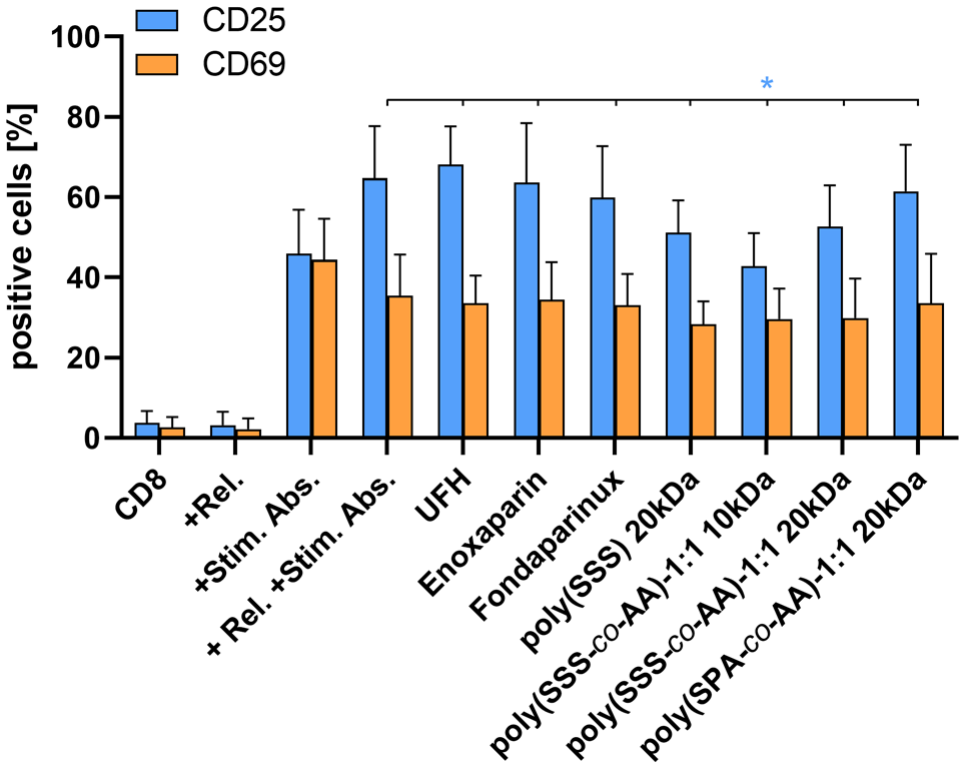
Inhibitory potential of heparin mimetics on the platelet releasate enhanced CD8^+^ T cell activation. Percentage of CD25 and CD69 expressing cells are shown for each investigated compound. Data represent means ± SD (*n* = 3) with *: *p* < 0.05

**Fig. 6:**
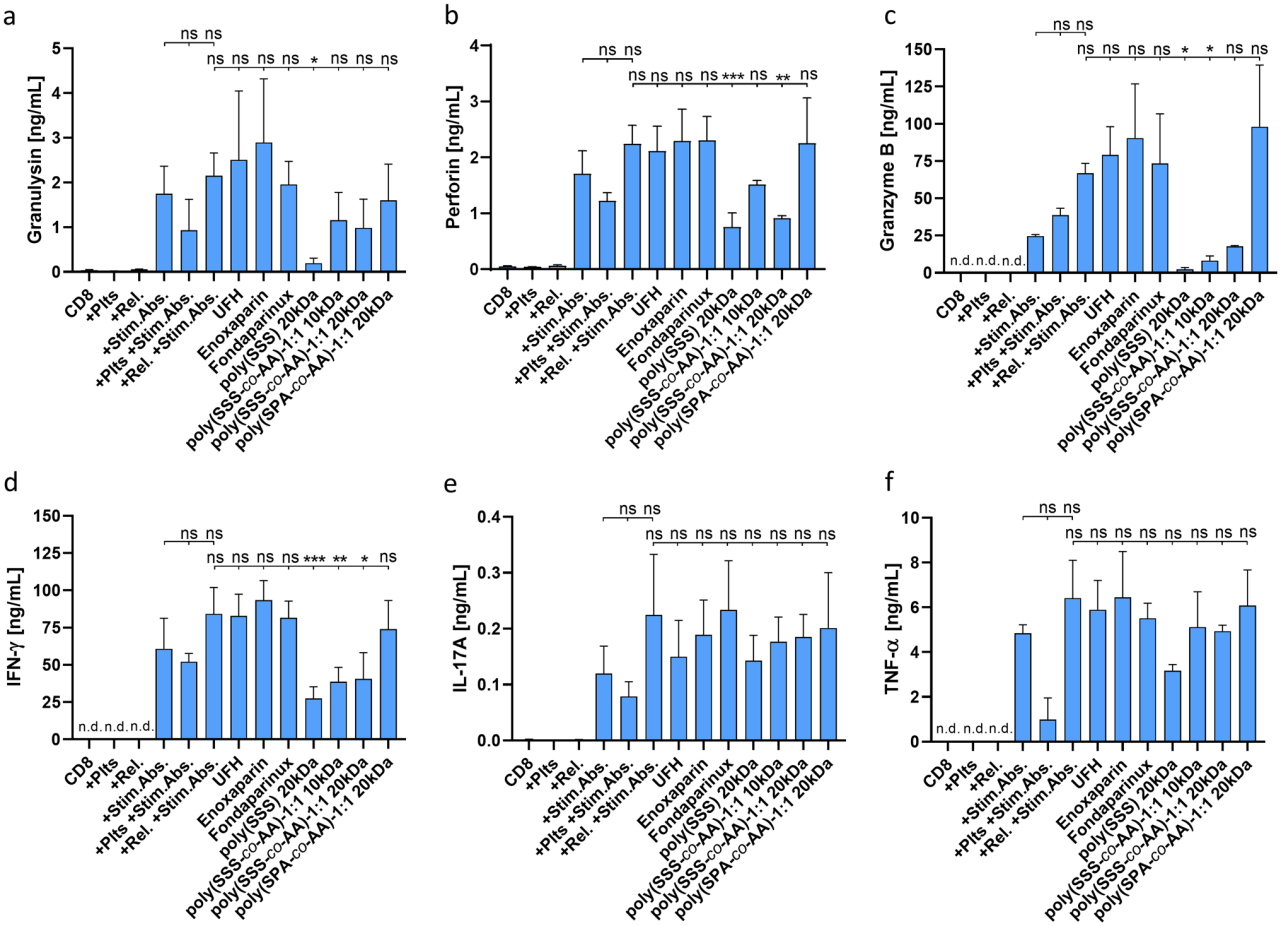
Release of cytolytic factors and pro-inflammatory cytokines from CD8^+^ T cells. Levels of **a** Granulysin, **b** Perforin, **c** Granzyme B, **d** IFN-*γ*, **e** IL-17A and **f** TNF-*α* were determined in supernatants of activated CD8^+^ T cells. T cells were either incubated with platelet releasates or samples treated with indicated compounds during tumour cell-induced platelet activation. Data represent mean ± SD (*n* = 3) with n.d.: not detectable, ns: not significant, *: *p* < 0.05, **: *p* < 0.01 and ***: *p* < 0.001

**Fig. 7:**
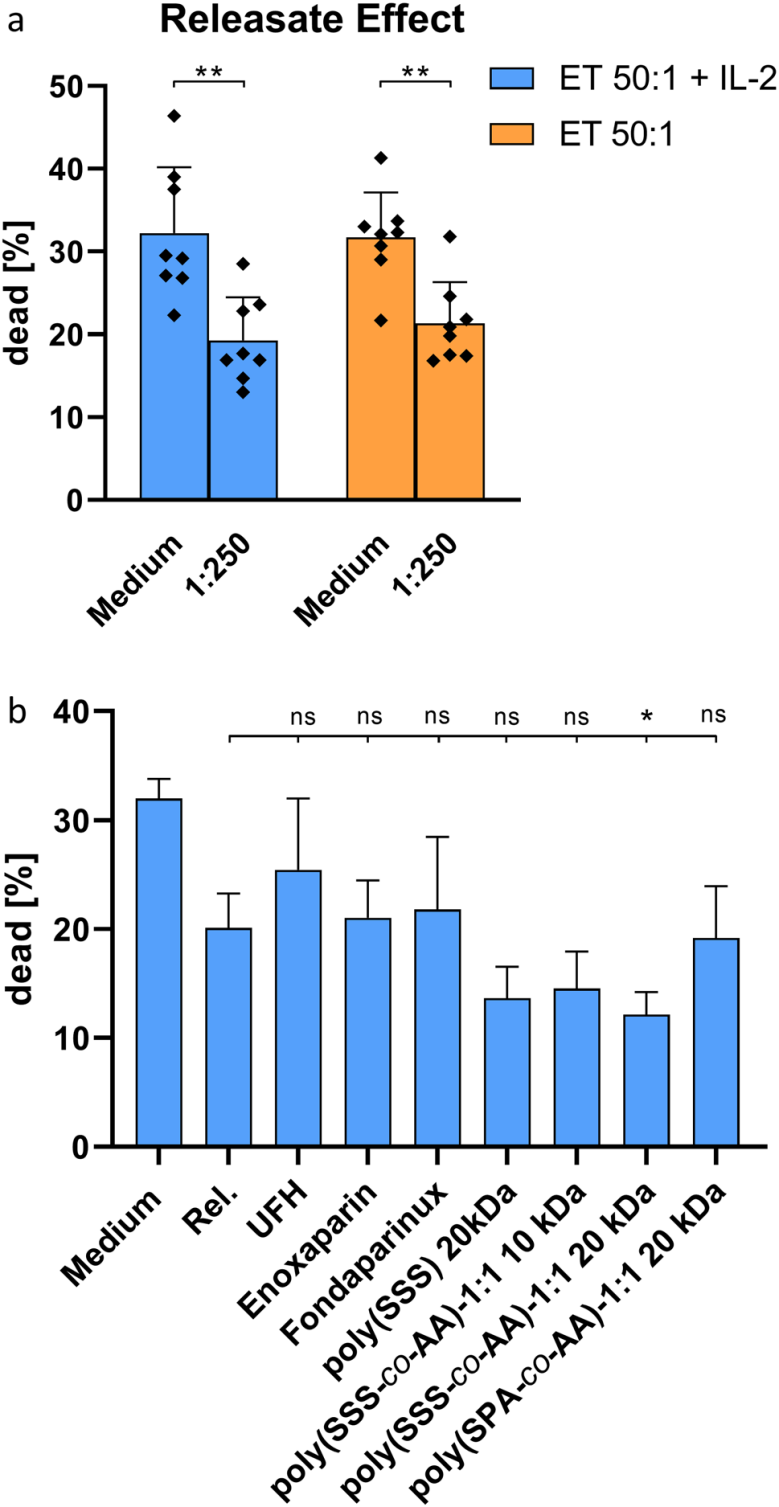
Assessment of cytolytic capacities of PBMC cultures against K562 target cells. **a** PBMCs were pre-incubated with platelet releasates in the presence or absence of IL-2. **b** Applied platelet releasates were prepared in the presence of heparin derivatives or heparin mimetics. Data represent mean ± SD of eight **a** and three **b** independent experiments, respectively. ns: not significant, *: *p* < 0.05 and **: *p* < 0.01

Data represent means ± standard deviations of at least three independently performed experiments (*n* = 3). GraphPad Prism Version 8 was used for statistical analysis. One-way ANOVA using Tukey post hoc analysis was conducted for data in Figs. 1, 2 and 3. Data in Figs. 4, 5, 6, and 7b were analysed by one-way ANOVA and Dunnett post hoc test. Figure 7a is analysed using students *t* test. ns: not significant; *: *p* < 0.05; **: *p* < 0.01; ***: *p* < 0.001.

## Results

### Release of IL-10 from PBMC is enhanced by platelets and their secreted mediators

IL-10 is considered as a key player in immune modulation and contributes to an immunosuppression [27, 28]. To evaluate a prospective immunosuppressive activity of platelets during haematogenous metastasis probably via inducing IL-10, we determined IL-10 levels from PBMC co-cultured with platelet and tumour cell-induced platelet releasates, respectively. In detail, PBMCs were activated with T cell-stimulating antibodies and co-cultured either with platelets or tumour cell-induced platelet releasates. While unstimulated PBMCs release only minor amounts of IL-10, more than twofold higher levels were detected in antibody stimulated samples reaching about 500 pg/mL. This secretion was massively enhanced by co-incubation with platelets (Fig. 1a) or tumour cell-induced platelet releasates (Fig. 1b). Since platelets hardly contained IL-10 themselves, this indicates their strong immunosuppressive activity. However, addition of external IL-2 did not lead to a further and significant elevation of IL-10 expression and release, neither in antibody stimulated samples, nor in stimulated samples co-cultured with platelets and releasates, respectively. Thus, we excluded IL-2 addition from further investigations.

### Platelet releasate-induced Treg differentiation is diminished by heparin and synthetic heparin mimetics

As an immunosuppressive CD4^+^ subset, Treg cells play a crucial role in cancer immune evasion. Based on the aforementioned results, we determined the impact of platelets and their releasates on the proliferation of Treg cells in an isolated human CD4^+^ T cell population, indicated as CD25/FoxP3 double-positive cells in flow cytometry (Fig. 2). Consistent with the IL-10 release, the activation with stimulating antibodies shifted the CD4^+^ cells to a regulatory phenotype. This shift was further enhanced by co-cultivation with platelets. Independent from the presence of whole platelets, platelet-derived mediators released upon tumour cell-mediated platelet activation were also sufficient to amplify the differentiation of CD4^+^ T cells to an immunosuppressive phenotype. Once more, when platelets or their releasates were added without stimulating antibodies, no induction of Treg cells was detected.

Heparin is known to possess various anti-tumour activities, and recent findings focussed platelets as promising targets of heparin or LMWH in the metastatic context. However, whether heparin can affect immunomodulatory activities of platelets remained completely open. To tackle this issue, we investigated heparin for their ability to alter the immunoinhibitory properties of tumour cell-induced platelet releasates (Fig. 3). Unfractionated heparin and LMWH enoxaparin reduced Treg differentiation slightly stronger than fondaparinux, which was previously shown to poorly interfere in tumour cell–platelet interactions [24]. Nevertheless, these reductions did not achieve significance. Recently, synthetic co-polymers consisting of either 4-styrenesulfonate (SSS) or 3-sulfopropyl acrylate (SPA) moieties co-polymerised with acrylic acid (AA) have been reported to efficiently affect tumour cell-induced platelet activation acting as promising heparin mimetics [25]. Notably, these mimetics were highly efficient and significantly inhibited Treg differentiation, outmatching the commercial heparins.

### Activation of CD8^+^ T cells by platelet releasates is impeded by heparin mimicking polymers

The impact of platelets on the activity of CD8^+^ effector T cells has previously been investigated with regard to various diseases. Notably, ambivalent effects representing either an activation or a deactivation of CD8^+^ cells were observed pointing out the complexity and treatment dependency of platelet effects on CD8^+^ T cells [14, 29, 30]. Since in a cancer-related model, tumour-induced activated platelets affect the immune balance into an immunosuppressive condition, we aimed to investigate whether they also affect CD8^+^ effector T cells in this direction. Therefore, we examined the expression of CD25 and CD69 on isolated CD8^+^ T cell cultures as markers for CD8^+^ T cell activity (Fig. 4). Concurring with the Treg data, sole addition of platelet preparations did not alter the ratio of CD25 and CD69 expression compared to unstimulated CD8^+^ T cells. As expected, the addition of a T cell-stimulating antibody cocktail led to a pronounced activation as indicated by elevation of CD25 and CD69 expression, respectively. Notably, although a 96 h incubation with platelet preparations led to a significant increase of CD25 expression, indicating a further activation, the CD69 levels were slightly diminished arguing against a clear upregulation of CD8^+^ T cells upon platelet stimulation. Of note, from preliminary experiments (data not shown), the variance between CD25 and CD69 seems not to originate from the expression kinetics of the early activation marker CD69.

Nevertheless, regarding the activation of CD8^+^ effector T cells, the commercial heparin formulations failed to reduce CD25 expression (Fig. 5). Concerning the heparin mimetics, an inhibitory tendency could be observed, while poly(SSS-*co*-AA)-1:1 10 kDa significantly reduced CD25 surface expression and thus cell activation. Again, CD69 levels did not change, neither in releasates nor in the heparin-treated preparations.

Since the modulation of CD8^+^ T cell activity remained rather ambiguous by solely focussing on surface marker expressions, we aimed to determine alterations in the cytolytic potential. Therefore, the release of pro-inflammatory and cytolytic factors from CD8^+^ T cell cultures was analysed (Fig. 6). Basically, a similar release pattern was observed for the investigated factors IFN-*γ*, Perforin, Granzyme B, Granulysin, IL-17A and TNF-*α*. Unstimulated CD8^+^ T cells displayed hardly any intrinsic release and were unaffected by platelet releasate incubation. Upon antibody stimulation, CD8^+^ T cells released their cytolytic and pro-inflammatory mediators. This release was further enhanced by the addition of platelet releasates. Interestingly, although platelet co-culture elevated CD25 levels, the release of CD8^+^ cell mediators was inhibited, underlining the ambivalence of the platelets CD8^+^ T cell modulation. Consistent with the CD25 expression, mediator release was mainly unaffected by the presence of the commercial anticoagulants during platelet releasate preparation. However, especially for the release of IFN-*γ*, Perforin as well as Granzyme B, the heparin mimetics containing 4-styrenesulfonate monomers showed remarkable inhibitory potential (Fig. 6).

### Heparin and heparin mimetics differentially affect releasate mediated NK cell alteration

NK cells represent an essential component of innate immunity and contribute to anti-tumour immune response. Thus, we investigated the modulating effect of tumour cell-induced platelet releasates on the cytolytic activity of NK cells against K562 target cells. As depicted in Fig. 7a, a 24 h pre-incubation of PBMCs with platelet releasates significantly reduced killing of K562 target cells by NK cells. However, the presence of IL-2 did not affect NK cell activity. The effect of platelet releasate was significantly reversed by the use of UFH, while enoxaparin and fondaparinux did not alter NK cell inhibition. Interestingly, NK cell activity was significantly lower after application of releasates prepared in the presence of heparin mimicking polymers containing 4-styrenesulfonate monomers indicating a certain, not yet elucidated effect of the polymers in this assay (Fig. 7b).

## Discussion

There is an increasing body of evidence that platelets, beyond their activities in haemostasis, possess an immunological competence affecting the immune system in diverse ways with respect to the pathological context. Although platelets are known to play a crucial role during haematogenous dissemination and metastasis formation, immunological functions of platelets therein remain elusive. Here, we show at a cellular in vitro level that platelets, after being activated by tumour cells, affect immune cells and generally shift them into an immunosuppressive phenotype. This is a further piece in understanding the rather tumour-supporting activities of platelets in metastatic spread. Moreover, heparin was shown to partly reverse these immunosuppressive functions of platelets. These findings, for the first time, assign heparin a targeted activity in affecting immune functions in oncology and fuel ongoing discussion on potential anti-tumour activities of heparin in a clinical setting.

In detail, co-cultures of PBMCs with whole platelets or tumour cell-induced platelet releasates enhanced the release of IL-10 indicating a shift towards an immunosuppressive environment. Corroborating this assumption, our data show that whole platelets, as well as tumour cell-induced releasates, evoked Treg differentiation from CD3/CD28/CD2 stimulated CD4^+^ T cells, although further cell types than regulatory T cells might contribute to IL-10 release from PBMCs. For instance, Gudbrandsdottir et al. showed that activated platelets can enhance the IL-10 secretion from monocytes [31]. However, our observation is consistent with previous findings by Gerdes et al. who showed that releasates of thrombin-activated platelets increase the Treg ratio in activated T cells [32]. Interestingly, Rossiant et al. recently showed that direct platelet contact is needed for IL-10 release from Treg cells, since supernatants from sCD40L-activated platelets were incapable of stimulating IL-10 release [16]. Differences in these findings might be caused by the varying stimuli used for platelet activation, which may lead to differential release of platelet granules or platelet extracellular vesicles, which were absent in our experimental setup (data not shown) [33].

Commercial heparins blocked the tumour cell-induced release of platelet granules and thus inhibited Treg differentiation. However, as in our previous investigations, the effects of the pentasaccharide fondaparinux were only weak, indicating that FXa inhibition alone appears not sufficient, and a need for glycosaminoglycan structure supports inhibitory effects, e.g. by attenuating cellular contacts [24, 25]. Regarding the synthetic heparin mimetics, the co-polymeric compounds effectively prevented Treg differentiation.

Concerning CD8^+^ T cell activation, analysis of CD25 and CD69 as activation markers in its reversion by heparin gave no explicit indication for inhibitory activation, while determination of CD8^+^ protein release resulted in clearer evidence. Interestingly, co-incubation with whole platelets or platelet releasates exerted contrary effects. While platelets clearly inhibited cytolytic release of activated CD8^+^ T cells, except for Granzyme B, tumour cell-induced platelet releasates further enhanced the activation with CD3/CD28/CD2 stimulating antibodies. Regarding the inhibitory capacity of the applied anticoagulants, all commercial heparins seemed rather inefficient to abrogate the releasate effect on CD8^+^ T cell secretion. Conversely, co-polymers containing a SSS moiety were more active than the heparins or the co-polymer consisting of SPA and AA, which is in line with our previous findings. Furthermore, as for Treg cell data, a divergent effect of platelet releasates obtained by different activation settings appears reasonable, since Rachidi and colleagues observed an inhibition of CD8^+^ T cell activity by thrombin-induced platelet releasates [34]. Cytotoxic CD8^+^ T cells are key players in anti-cancer immunity. Thus, an upregulation of CD8^+^ T cell cytolytic activity would be considered disadvantageous for cancer progression, which in turn results in a negative effect of interfering in platelet release for cytotoxic t-Lymphocyte-mediated tumour cell clearance. However, it remains elusive whether platelets’ inhibitory or releasates’ activating effect would outweigh each other in an in vivo approach.

More clearly and convincingly, the cytolytic activity of NK cells was determined as another essential component of anti-cancer immunity. In line with previous findings, our tumour cell-induced platelet releasates reduced the activity of NK cells against K562 target cells [7, 35]. Since UFH seemed to reverse the attenuated NK cell activity induced by platelet releasates, this effect might be a further explanation and argument for the potential anti-metastatic activity of heparin. Regarding the SSS containing heparin mimetics, it was rather surprising to detect an additional reduction of NK cell activity instead of a recovery, since we observed a strong inhibition of mediator release from tumour cell-activated platelets in our previous study [25]. However, this observation might be caused by an intrinsic immunomodulatory capacity of the SSS-containing polymers towards NK cells, which has to be further evaluated in future studies.

In conclusion, this study shows the potential of tumour cell-induced platelet releasates to modulate a broad range of components involved in anti-cancer immunity into a generally immunosuppressive state, providing further arguments for the metastasis-supporting activities of platelets. Heparin, i.e. LMWH, clinically established in the oncological sector might be promising in antagonizing these platelet effects. As one of the major mechanisms of interference, we postulate the inhibition of platelet activation that we showed for heparins and heparin mimetics in our previous studies [24, 25]. On a molecular basis, RANTES, PF4, and TGF-*β* appear as relevant mediators to be involved in the regulatory T cell differentiation or alteration of CD8^+^ and NK cell activity [7, 34, 36]. Since heparin is known to possess a binding capacity for a variety of chemokines [37], a binding of those molecular effectors by heparin or the mimetics might alter their biological activity and would give rise to a further possible mechanism of interference that has to be evaluated in future studies. Thus, this study provides first insights into the interplay of platelets, the cellular immunity and heparins for a deeper understanding of postulated anti-tumour activities of heparin beyond their anticoagulant efficiency.

### Supplementary Information

Below is the link to the electronic supplementary material.

Supplementary file1 (PDF 791 KB)

## Data Availability

Research Data and Material are available from the corresponding author on reasonable request.

## Abbreviations

AA: Acrylic acid
plts: Platelets
rel.: Platelet releasates
SPA: Potassium-3-sulfopropyl acrylate
SSS: Sodium 4-styrenesulfonate
