# Supplementary material for: Tumour cell-activated platelets modulate the immunological activity of CD4+, CD8+, and NK cells, which is efficiently antagonized by heparin
Source: Cancer Immunol Immunother. 2022 Mar 14;71(10):2523–33. doi: 10.1007/s00262-022-03186-5 (PMC9463253; doi:10.1007/s00262-022-03186-5)
Supplement: Supplementary file 1 — Supplementary file1 (PDF 791 KB) [file 262_2022_3186_MOESM1_ESM.pdf]

Supplementary materials

Cancer Immunology, Immunotherapy (submitted in 2021) – Lukas M. Gockel et al.

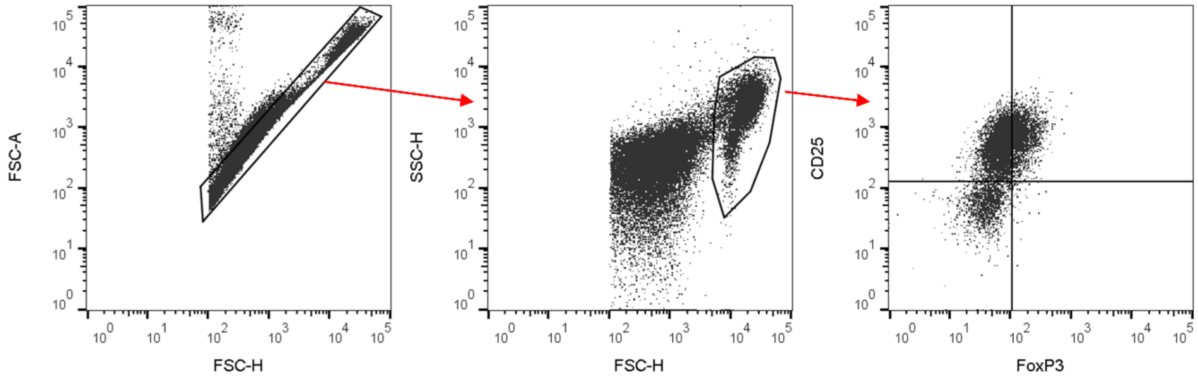

Supplementary Figure 1 Gating strategy for identification of regulatory T cells depicted at a sample of CD4$^+$ T cells activated by stimulating antibodies and platelets. Isolated CD4$^+$ T cells were gated for singlets by FSC height vs. area. Subsequently cells of interest were selected by FSC vs. SSC gating to exclude platelets and debris from analysis. Finally, Tregs were identified as FoxP3 and CD25 double positive population by use of compensation and FMO controls

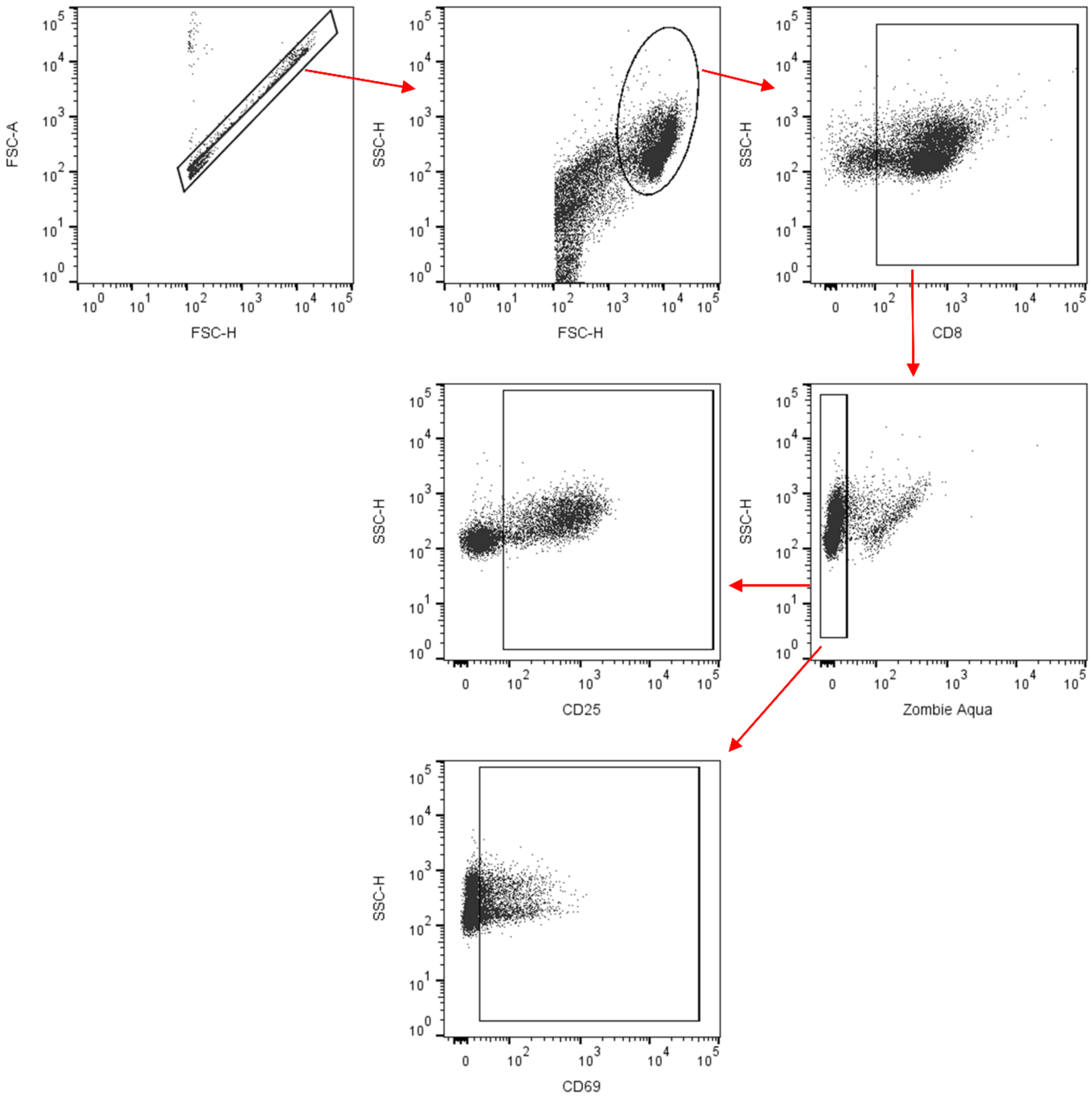

Supplementary Figure 2 Gating strategy for identification of activated CD8 T cells is depicted at a sample of $CD8^+$ T cells activated by stimulating antibodies and platelet releasate. Acquired cells were considered as singlets by gating FSC height vs. area. Following, cells of interest were selected by FSC vs. SSC gating. Viable CD8 cells were identified by gating CD8 positive events and subsequent exclusion of dead cells by viability dye Zombie Aqua. The viable population was analysed for expression of CD25 and CD69. All gating steps were performed by use of compensation and FMO controls

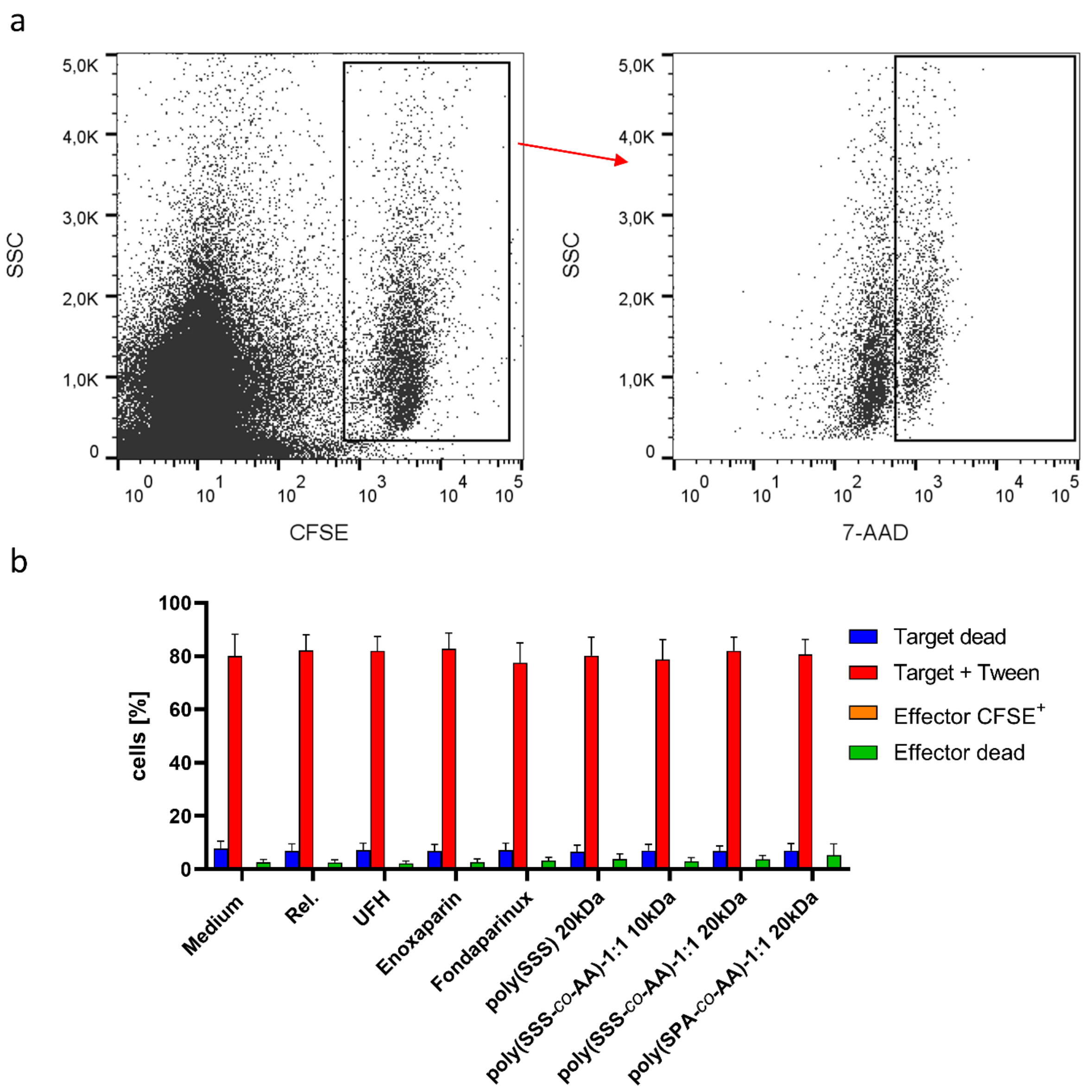

Supplementary Figure 3 (a) Gating strategy for identification of dead K562 target cells depicted at a ET 50:1 sample. K562 cells were identified as CFSE positive cells and determined as dead cells by viability dye 7-AAD. (b) As controls dead cells were determined for K562 target cells alone, PBMC effector cells alone, and Tween treated target cells. Further, by gating for $CFSE^+$ cells in the effector cells only sample it was assured that the gate was set up correctly and no false positive events were included to the analysis
